# Quantifying distortions in two-photon remote focussing microscope images using a volumetric calibration specimen

**DOI:** 10.3389/fphys.2014.00384

**Published:** 2014-10-08

**Authors:** Alexander D. Corbett, Rebecca A. B. Burton, Gil Bub, Patrick S. Salter, Simon Tuohy, Martin J. Booth, Tony Wilson

**Affiliations:** ^1^Department of Engineering Science, University of OxfordOxford, UK; ^2^Department of Physiology, Anatomy and Genetics, University of OxfordOxford, UK

**Keywords:** remote focussing microscopy, cardiac imaging, distortion

## Abstract

Remote focussing microscopy allows sharp, in-focus images to be acquired at high speed from outside of the focal plane of an objective lens without any agitation of the specimen. However, without careful optical alignment, the advantages of remote focussing microscopy could be compromised by the introduction of depth-dependent scaling artifacts. To achieve an ideal alignment in a point-scanning remote focussing microscope, the lateral (XY) scan mirror pair must be imaged onto the back focal plane of both the reference and imaging objectives, in a telecentric arrangement. However, for many commercial objective lenses, it can be difficult to accurately locate the position of the back focal plane. This paper investigates the impact of this limitation on the fidelity of three-dimensional data sets of living cardiac tissue, specifically the introduction of distortions. These distortions limit the accuracy of sarcomere measurements taken directly from raw volumetric data. The origin of the distortion is first identified through simulation of a remote focussing microscope. Using a novel three-dimensional calibration specimen it was then possible to quantify experimentally the size of the distortion as a function of objective misalignment. Finally, by first approximating and then compensating the distortion in imaging data from whole heart rodent studies, the variance of sarcomere length (SL) measurements was reduced by almost 50%.

## Introduction

Remote focussing microscopy is a technique that enables optically sectioned, in-focus images of a specimen to be taken outside of the focal plane of the imaging objective. In principle, any microscope that achieves refocussing by modifying the optical phase at the back aperture of the imaging objective can be considered a remote focussing microscope. Approaches described in the literature include acousto-optic deflectors (Duemani Reddy et al., 2008; Kirkby et al., 2010), spatial light modulators (Abrahamsson et al., 2013; Jesacher et al., 2013), and electrically tuneable lenses (Grewe et al., 2011; Jabbour et al., 2014).

The remote focussing technique explored in this paper uses a reference objective to axially displace the focal spot (Botcherby et al., 2007, 2008a,b; Salter et al., 2009). In conventional microscopes, illuminating the objective with defocussed light to illuminate points outside of the focal plane generates strong spherical aberrations, resulting in a blurry image. In a remote focussing microscope, these aberrations are pre-compensated using a matched “reference” objective. This is a significant development as it allows images of the entire specimen volume to be acquired without the need to move either the specimen or the objective. Moreover, the ability to scan over large depth ranges (±100 μm) can be achieved at speeds equivalent to lateral (XY) scan speeds, allowing fast events in excitable cell networks to be captured. These attractive properties have led to the adoption of remote focussing microscopy by a number of groups around the world (Anselmi et al., 2011; Hoover et al., 2011; Kumar et al., 2011).

To be a valuable tool in the biomedical sciences, images acquired with remote focussing microscopes would ideally have a sufficient fidelity to allow quantitative measurements of anatomical structure to be made. This is especially true when extracting anatomical information from optical images of cardiac tissue, containing finely spaced (2 μm) sub-cellular sarcomere structures. The small natural variability of the sarcomere length (SL) mean that image distortions of even a few percent across the field of view can imply large differences in cell physiology. To make reliable, repeatable measurements from microscopy data requires a new set of calibration tools, which can validate the accuracy to which distance, area, and volume measurements can be made. This paper explores the application of one such tool for measuring and correcting objective misalignment by monitoring the impact of axial displacement on the shape of the three-dimensional imaging volume.

The basic components of a point scanning remote focussing microscope are a light source, a set of lateral (XY) scan mirrors, a “reference” objective focussed on an axial (Z) scan mirror and an imaging objective. Once collimated, the light source illuminates the XY scan mirrors, which are then imaged onto the back focal plane of the reference objective. The Z scan mirror sends light back through the reference objective toward the imaging objective. The position of the Z-scan mirror determines the axial displacement of the focal plane within the specimen (see Botcherby et al., 2012 for more details). The back focal plane of the reference objective is imaged onto the back focal plane of the imaging objective. To ensure complete cancelation of spherical aberration, there must be unit magnification between the reference and imaging objectives. The precise alignment of this optical train requires exact knowledge of the location of the back aperture of the reference and imaging objectives, which for most commercial objectives, is unavailable.

In simple objectives, the back focal plane includes a physical aperture and is located one focal length behind the imaging lens. This ensures that rays passing through the center of the physical aperture (the chief rays) will arrive normal to the focal plane. In this configuration the lens is said to be telecentric and the magnification will be independent of focus (Stelzer, 2006). More complex objective lenses consist of multiple lens elements and the position of the physical aperture is not always clear. The commercial sensitivity of the objective lens design means that the distribution of lenses and apertures within the barrel cannot be easily determined.

In this paper, we take an empirical approach to determining the ideal objective alignment in a remote focussing microscope by imaging a volumetric calibration specimen. As the structure of the calibration specimen is known throughout the volume, it is possible to quantify imaging distortions associated with misalignment of the objective lens. By measuring image distortion for a few objective positions it is possible to interpolate between these locations to obtain an ideal objective alignment and ensure telecentric operation with minimal distortion.

## Materials and methods

### Microscope simulation

**Figure 1:**
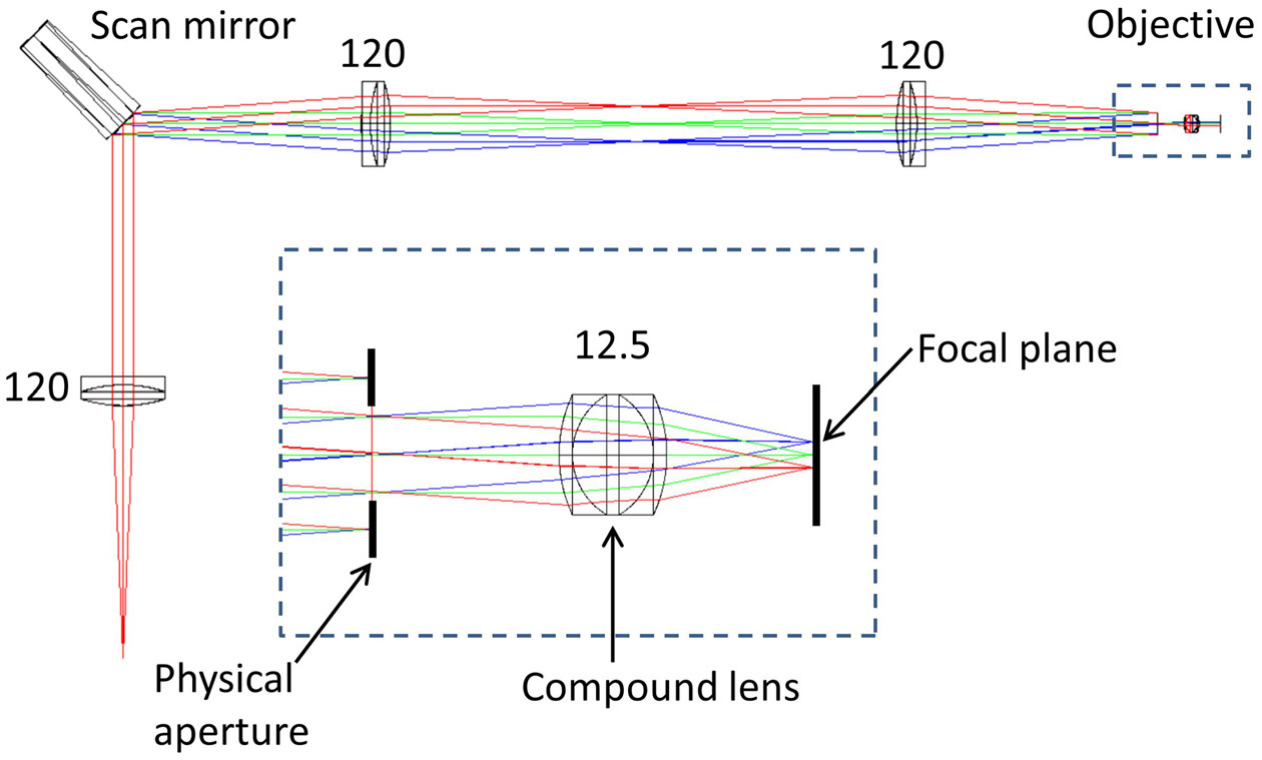
**ZEMAX optical model of a simplified scanning microscope**. The rays are color coded according to the off-axis tilt angle of the lateral (X) scan mirror; red = +2°, green = 0°, blue = −2°. Inset is shown the model of an imaging objective, composed of a triplet lens and a physical aperture. Numbers indicate focal lengths in millimeters. See text for full details.

To determine the impact of axial and lateral misalignment of the objective, simulations were performed in ZEMAX (ZEMAX Development Corporation). The model used is outlined in Figure 1. In this model the laser source is first collimated before illuminating a lateral scan mirror (for simplicity, this is single axis only). Using the ZEMAX multi-configuration tool it was possible to visualize the effect of multiple scan angles in the same model. The scan angles of −2°, 0°, and +2° were chosen to match the scan angles in the experimental system (see Section Remote Focussing Microscopy). As an optical model for the commercial experimental objective was unavailable, a simplified triplet lens (*f* = 12.5 mm) with an 8 mm entrance aperture was used. The inset in Figure 1 shows the image of the scan mirror coincident with the physical aperture of the objective model. This is the ideal alignment configuration as rays passing through the center of the physical aperture (chief rays) are steered by the lens to arrive normal to the focal plane. The purpose of the model was to identify the change in the geometry of the rays illuminating the specimen with displacements of the objective lens.

### Remote focussing microscopy

**Figure 2:**
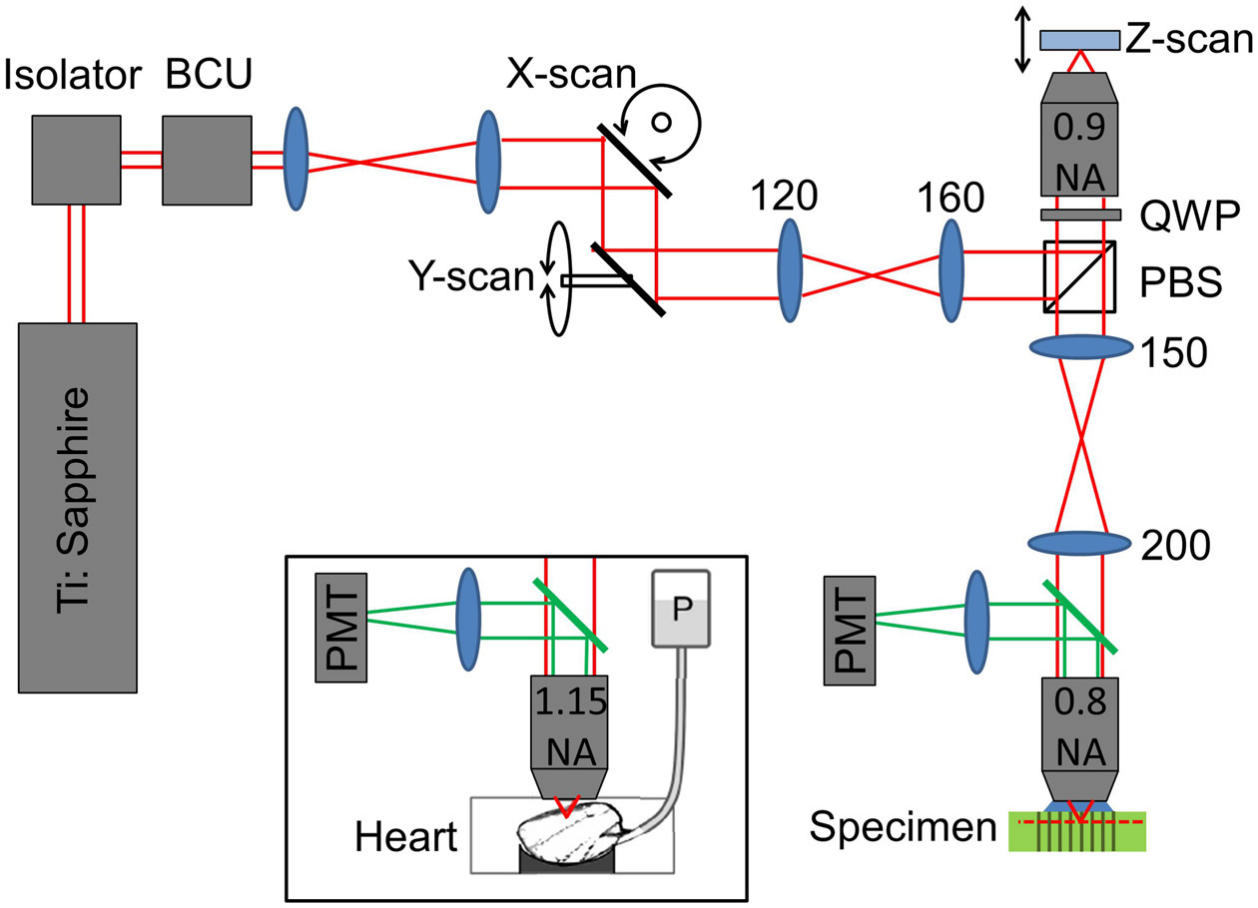
**Sketch showing the optical layout of the remote focussing microscope (see main text for details)**. Where shown, numbers indicate lens focal lengths in millimeters. BCU, beam conditioning unit; PBS, polarizing beam splitter; QWP, quarter wave plate; PMT, photomultiplier tube; P, gravity fed perfusion of the cardiac tissue.

To validate the predictions of the ZEMAX model, a remote focussing microscope was used together with a volumetric calibration specimen (described below). The optical layout of the remote focussing microscope is shown in Figure 2. Light from a pulsed Ti:Sapphire source (Tsunami, SpectraPhysics; 80 MHz, 850 nm) is first passed through a Faraday isolator and into a custom built beam conditioning unit (BCU) which controls the laser intensity. A telescope expands the beam onto a pair of closely spaced lateral (XY) scanning galvanometers. The scan mirrors are then imaged onto the back focal plane of the first reference objective lens (Olympus UApo/340, 40×, 0.9 NA dry objective). The reference objective focuses the light onto a small mirror, which can be moved axially by a pair of coupled galvanometers. A quarter wave plate ensures maximum transmission through the polarizing beam splitter and into the microscope head (an upright Olympus BX60M). A relay telescope images the back focal plane of the “reference” objective onto the back focal plane of the imaging objective (Olympus LUMPlanFL N 40×, 0.8 NA water dipping objective). As Olympus lenses are designed to operate with 180 mm focal length tube lenses, the reference objective has a magnification of *M*_1_ = 40 × (200/180) = 44.44, and the imaging objective has a magnification of *M*_2_ = 40 × (150/180) = 33.33. Accounting for the different refractive indices of the immersion media (*n*_1_ = 1, *n*_2_ = 1.33), the magnification between conjugate image planes is then given by *M*_SYS_ = (*n*_2_*M*_2_/*n*_1_*M*_1_) = 1. Finally, the imaging objective tightly focuses the pulsed source into the specimen, generating two-photon fluorescence. The fluorescence light is captured by the imaging objective and filtered using a dichroic mirror (Semrock - FF568-Di01-25x36) before being detected using a photon counting PMT (Hamamatsu H7422P series).

### Cardiac imaging

To identify the impact of objective misalignment on the imaging volume, image stacks of intact heart tissue were used. Imaging of the cardiac tissue was conducted using the same optical setup shown in Figure 2 with the exception that the imaging objective was exchanged for an Olympus UAPO W340 40×, 1.15 NA lens, (working distance, 250 μm) to help resolve the fine sarcomere structures (see Figure 2 inset). The back aperture of the objective was illuminated with a 90 mW beam via a dichroic fold mirror (Chroma 720dcspxr coating on a Linos blank, not shown in Figure 2). Backscattered excitation light was filtered out from the two-photon epi-fluorescence signal by passing back through the dichroic fold mirror. The fluorescence light was filtered once more by a 575–650 nm bandpass filter (Semrock - FF568-Di01-25x36) before being detected by the Hamamatsu photon counting PMT.

For the tissue preparation, all procedures conformed to the Animals (Scientific Procedures) Act 1986 (United Kingdom). Three strains were used [Sprague-Dawley (SD), Wistar-Kyoto (WKY), and spontaneously hypertensive rats (SHR)], but results are treated as 1 group for the purpose of this study. In brief, hearts were swiftly isolated from female rats (≈250–300 g) killed by an overdose of anesthetic (pentobarbital), rinsed in ice-cold normal Tyrode buffer, and the aorta was swiftly cannulated for coronary perfusion in Langendorff mode. To prevent intra-ventricular fluid accumulation, an incision into the pulmonary artery wall just past the right ventricular outflow valve was performed. Further details on the methods can be found in Botcherby et al. (2013).

Whole hearts were dye loaded by coronary perfusion for 5 min with 5 μM di-4-ANEPPS (Invitrogen) in normal Tyrode using a micro-injector. Di-4-ANEPPS is a membrane-bound dye which reveals the structure of the t-tubules. Di-4-ANEPPS was chosen for its low toxicity and strong multi-photon response. Hearts were then placed on a bespoke cradle and gently stabilized by a nylon mesh placed on top of the tissue. For imaging, hearts were cardioplegically arrested at room temperature. Before imaging, the objective was lowered as far as possible using the focus dial so that the tip of the objective was in contact with the surface of the heart tissue.

### Specimen fabrication

**Figure 3:**
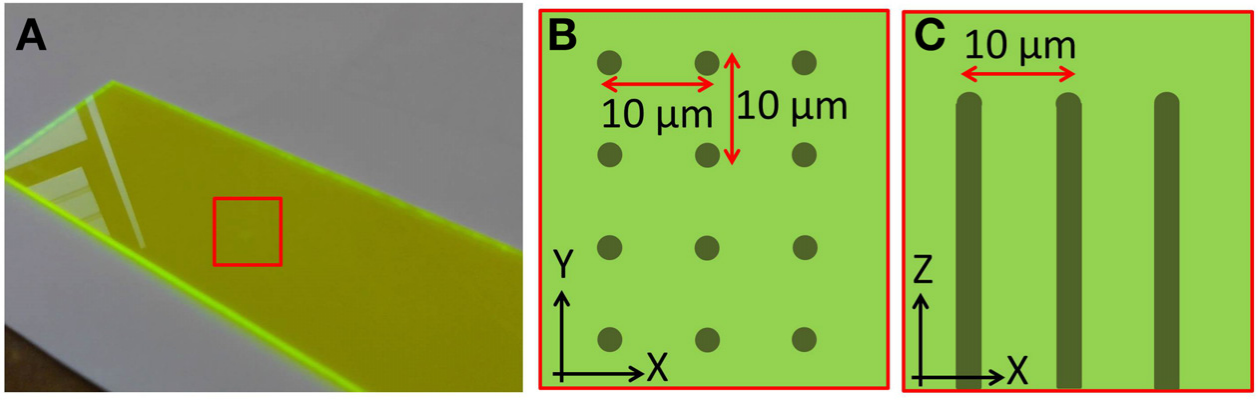
**Images showing the structure of the laser fabricated calibration specimen**. **(A)** Macroscopic image of the plastic dye-doped substrate. The fabrication region is indicated by the red square. Schematics showing how points were fabricated on a 10 μm square grid **(B)** over a 500 × 500 μm area, and extended axially **(C)** over a depth of 200 μm.

The calibration specimen used to align the remote focussing microscope contains regularly spaced features that allow subtle distortions of the imaging volume to be identified. The calibration specimen consisted of a regular array of columnar structures fabricated using laser writing within a dye-doped plastic substrate (part no. 92001, Chroma Technology Corp.). The fabrication pattern consisted of a 10 μm square array of points in the XY plane. Repeated fabrication steps extended the points vertically throughout the specimen to form columns 200 μm deep, starting 20 μm below the specimen surface (Figure 3). The pattern extended over 500 μm laterally, overfilling the field of view of the microscope. The width of each column was approximately one micrometer.

Laser fabrication of the calibration specimen was achieved using an amplified Ti:Sapphire laser (Solstice, Newport Spectra-Physics; 100 fs pulses, 1 kHz repetition rate, 800 nm) which was tightly focussed into the plastic substrate. Non-linear absorption arising from the ultrashort pulses resulted in melting of the plastic substrate. The non-linear nature of the process caused any structural modification to be limited to the focal volume of the fabrication beam. Fabrication required adaptive phase correction to compensate for spatially-varying aberrations when fabricating deep within the specimen. See Jesacher and Booth (2010) and Salter et al. (2014) for more details of the adaptive fabrication process.

### Image processing

**Figure 4:**
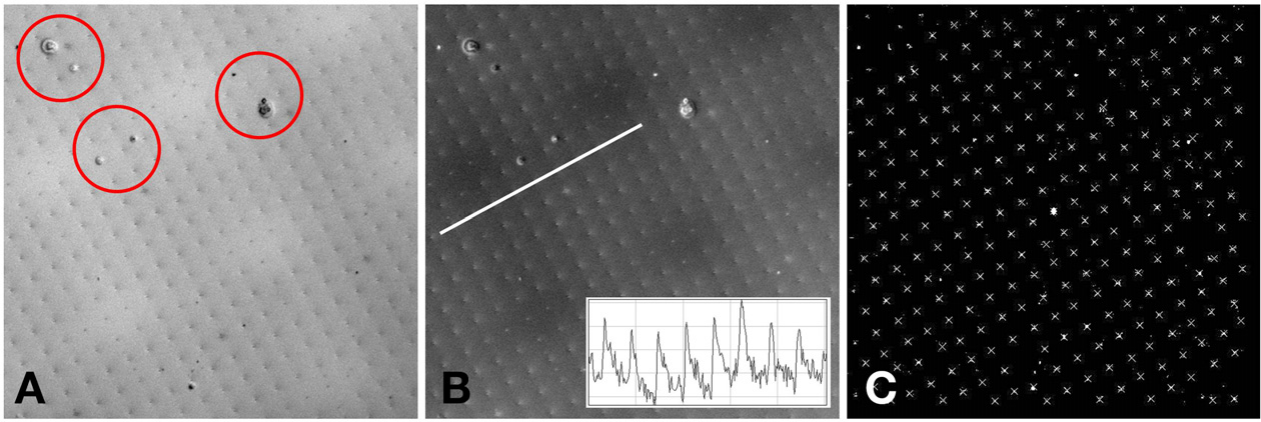
**(A)** Example two-photon image of the fluorescent specimen. Flaws in the plastic substrate are shown encircled. **(B)** Inverted version of **(A)**. A line profile through the image (inset) shows the signal to noise ratio of the fabricated features. **(C)** Processed version of **(B)**. The white crosses indicate the centroids of the local maxima. The white diamond shows the center of the 512 × 512 image. The separation between the fabricated points is 10 μm.

The raw two-photon images of the fluorescent specimen show a bright field, with dark fabrication features (Figure 4A). In addition to the fabricated features, there were several larger scale specimen flaws within the volume of the plastic substrate (shown encircled in Figure 4A). The acquired two-photon images were first inverted to identify the fabrication features as local bright points. The line transect inset in Figure 4B shows typical levels of signal to noise that can be achieved using this specimen.

To determine the precise location of these bright points, a semi-automated approach was taken. First, the whole image was thresholded to the brightest 1% of pixels. The value of 1% was chosen as the fabrication features are 1 μm in size and spaced by 10 μm, therefore the area covered by the bright features (in the inverted image) is approximately 1%. The thresholded image was used as a guide for the user to identify four points that make up a single unit cell of the square point array. The raw inverted image was windowed about these four points and the centroid was calculated from the brightest 1% of pixels within the window. The four centroids were then used to identify the lattice vectors of the square array. From a starting point defined by the user, the current point of interest was automatically incremented using the calculated lattice vectors until the whole image had been processed (Figure 4C). At each new lattice node a window was drawn and the centroid was calculated for the brightest 1% of pixels within the window. By setting all values below threshold to zero, it avoided background pixels biasing the centroid toward the center of the window. To ensure that only one fabrication point appeared within the image window, the window size was set to be 80% of the smallest lattice vector magnitude.

## Results

### Simulated lens displacement

**Figure 5:**
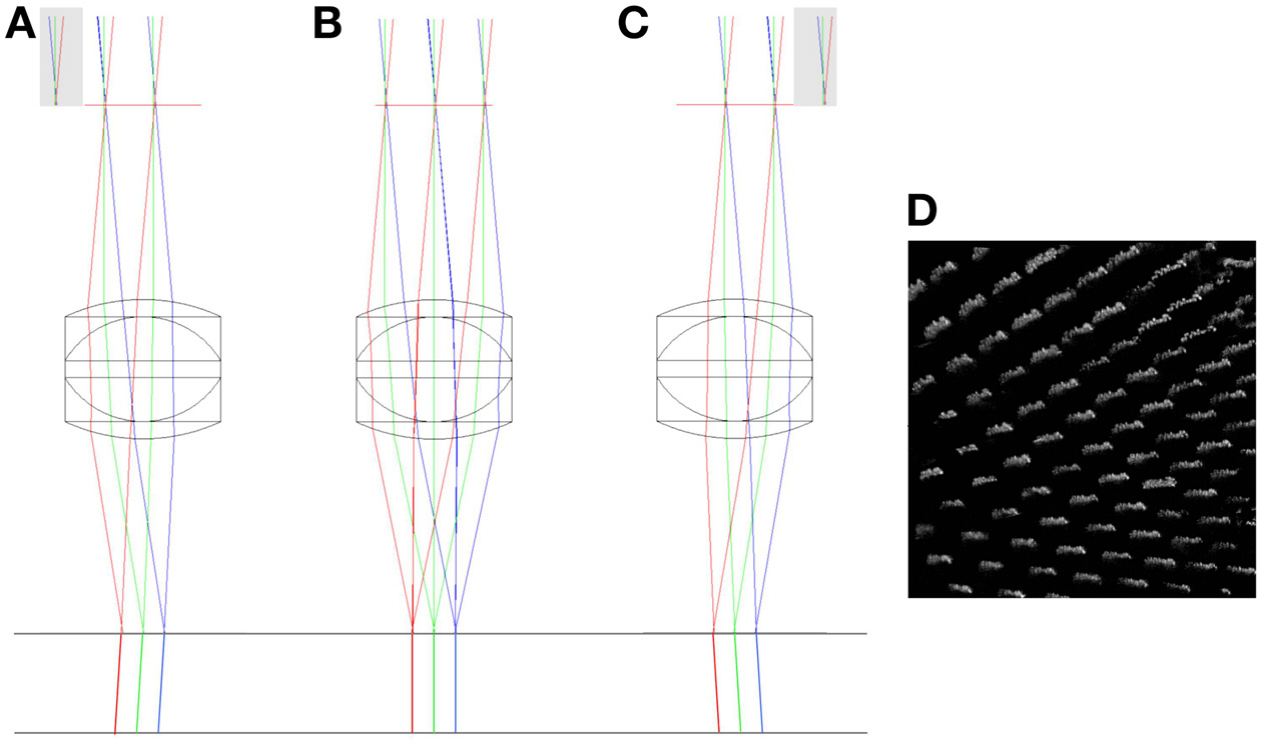
**The impact of lateral misalignment on specimen illumination**. The top horizontal line shows the focal plane of the triplet (*f* = 12.5 mm) objective. The chief rays have been extended below the focal plane to highlight the change in direction when the objective is displaced 2 mm to the left **(A)**, when it has zero lateral displacement **(B)** and when it is displaced 2 mm to the right **(C)**. Gray regions indicate clipping of the scan mirror image by the physical aperture. A simulation of the effect of the lateral displacement in **(C)** on a volumetric image of the columnar specimen is shown in **(D)**. See main text for full details.

By simulating the lateral and axial displacements of the imaging objective, it is possible to predict the impact of the displacements on the imaging volume. Actual distortions of the imaging volume measured using the calibration specimen can then be related back to these predications to identify their origin. A pure lateral (XY) displacement of the objective lens relative to its ideal location has two main effects. The first is that the chief rays leaving the objective are no longer normally incident to the focal plane, but are inclined at a small angle to normal (Figures 5A–D). For small objective displacements, there is a linear relationship between the size of the displacement and the angular deviation of the beam (Hecht, 1997). When imaged with a laterally offset objective, the calibration specimen will appear to be viewed off-axis (Figure 5D).

The second effect is that the displacement of the physical aperture clips the image of the XY scanning mirror, reducing the illumination of the specimen. This vignetting of the beam reduces the power that can be focussed into each spot and hence the multi-photon fluorescence efficiency. As can be seen in Figure 5, the separation of the focal points created by the extreme positions of the scan mirror (red and blue rays) remains the same for each configuration. This shows that there is no significant change in the magnification of the specimen with lateral displacement of the objective.

**Figure 6:**
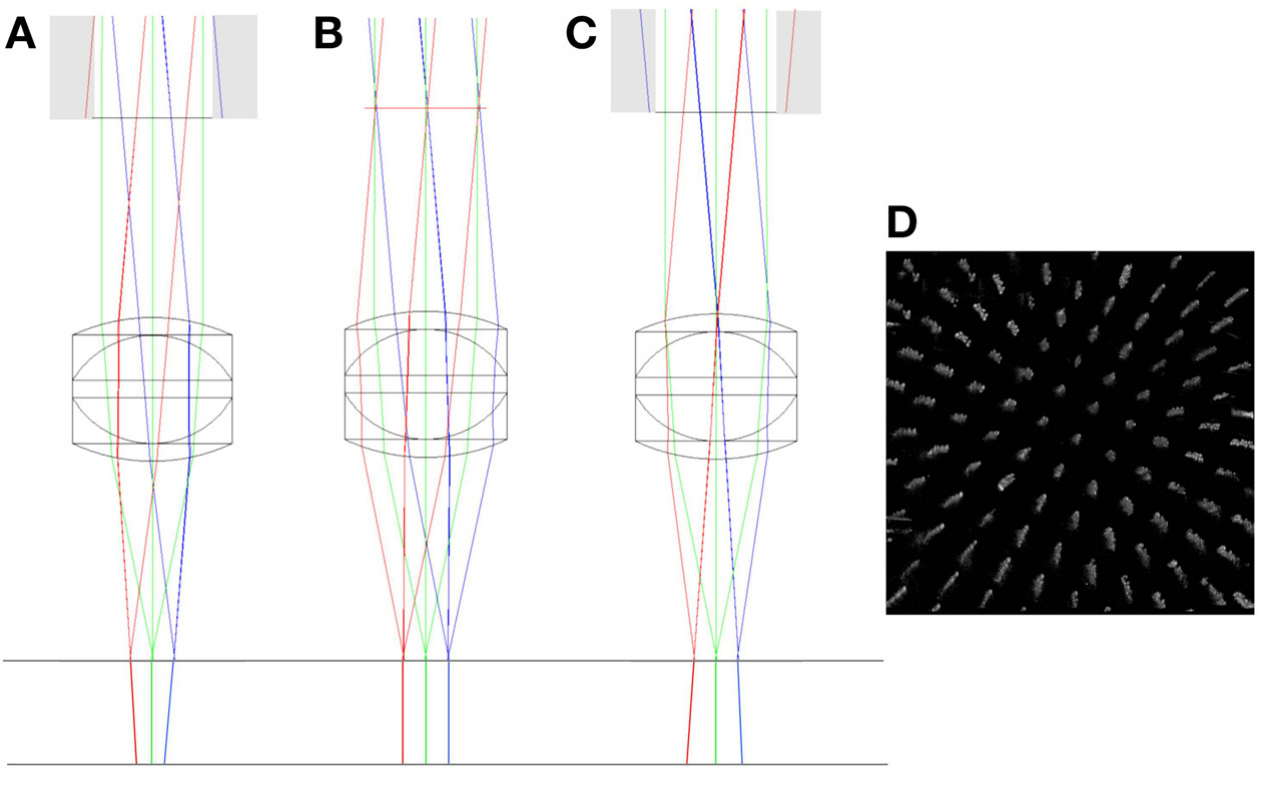
**ZEMAX drawings showing the change in the vergence of chief rays with objective misalignment**. The top horizontal line shows the focal plane of the triplet (*f* = 12.5 mm) objective. The chief rays have been extended below the focal plane to highlight the change in vergence. The simulations show axial misalignment of 10 mm below focus **(A)**, at focus **(B)**, and 10 mm above focus **(C)**. Gray regions indicate clipping of the scan mirror image by the back aperture. A simulation of the effect of the axial magnification in **(C)** on a volumetric image of the columnar specimen is shown in **(D)**. See main text for full details.

Large axial displacement of the lens also leads to vignetting of the beam as rays from edges of the scan mirror image no longer pass through the physical aperture of the objective (shown as gray regions of Figure 6). In addition to this, the chief rays originating from the scan mirror image no longer run parallel to the optic axis after passing through the objective, but will either diverge (scan mirror image inside focus) or converge (scan mirror image outside focus). This leads to a depth-dependent change in magnification (Figure 6). A pencil of rays emerging from the scan mirror image will still form a tight focus as before (and in the same location), the difference being that the center of focus will now shift laterally either side of the focal plane. This effect is often used as a test for the loss of telecentricity (Stelzer, 2006), by tracking the lateral displacement of fluorescent beads with defocus.

Depth-dependent changes in magnification only present a problem when imaging outside of the focal plane. Typically, in confocal and two-photon imaging, the focal plane remains fixed and the specimen is translated through the focal plane. In this case, small amounts of non-telecentricity do not lead to a distortion of the captured image stack. However, in multi-photon remote focussing microscopy, the specimen remains fixed and two-photon fluorescence light is captured from points above and below the focal plane. In this case the depth-dependent magnification leads to a significant distortion of the imaging volume with the degree of distortion being a sensitive function of the objective alignment. Whilst we focus here on remote focussing using a reference objective, the same distortions would apply to any other remote focussing methods that acquire information outside of the focal plane.

### Remote focussing data

In order to determine the impact of objective displacement on imaging volume distortion, images of the calibration specimen were collected. The calibration specimen was composed of 1 μm features on a 10 μm square grid, extending over 200 μm in depth (see the materials and methods section for further details). Three sets of images of the calibration specimen were obtained, each set corresponding to a different axial location (*Z*_0_-value) of the objective. The axial locations of the objective were measured relative to the position obtained by imaging the top surface of the reference objective barrel onto the top surface of the imaging objective barrel (position *Z*_0_ = 0 mm). Two other axial locations were determined by the limit of travel of the focussing dial of the microscope head. These axial locations were *Z*_0_ = −7 mm (i.e., moving 7 mm further away from the reference objective) and *Z*_0_ = +15 mm. For each *Z*_0_ position, 11 images were taken. These images consisted of a focal plane image, plus five images above and five images below the focal plane position. The images were taken in steps of 10 μm covering a total range of 100 μm. Each stack of 11 images was processed as described above to identify the fabrication point locations and their average lateral displacement. The fabrication point locations were tracked throughout the stack of images. If a point became “lost” due the presence of a flaw in the substrate, it was discarded and did not contribute to the measurement of the average lateral point displacement.

Due to the bulk loading of the plastic substrate with dye, relatively low excitation powers were required to image the specimen (<70 mW at the back aperture of the objective). Using a commercial transmission microscope (Axioplan 2, Carl Zeiss AG), the upper surface of the fabrication structure could be determined to be parallel (within the tolerance of the stage alignment) to the surface of the plastic substrate. However, when imaging with the remote focussing microscope, there remained a significant inclination of the microscope stage that could not be accurately corrected. The limited precision was due to the microscope stage being supported by four posts, each of which must each be adjusted manually to obtain a new stage height. The inclination of the microscope stage rotates the three-dimensional (3D) reconstruction of the calibration specimen in the same way as a lateral objective misalignment and the two effects could not be decoupled. For the purposes of identifying the efficacy of the calibration specimen, the experiment was restricted to accurately identifying the ideal axial displacement of the objective lens using the depth-dependent magnification change. The apparent tilt of the specimen from the lens/stage combination led to a displacement of the square array pattern between adjacent images in a 3D stack. These displacements were sufficiently small to allow them to be compensated in post-processing, effectively nulling the specimen tilt and leaving only the depth-dependent magnification information.

### Change in magnification with depth

In order to determine the change in image magnification as a function of depth in the specimen, the lateral spacing between the columnar features of the specimen was used. The unit cell of the square array created by the column features is known to be 10 μm. Any change in the apparent size of the lateral separation in the processed images will then be a consequence of the change in the physical field of view with depth in the specimen. The lateral separation appears to change as the distance calibration calculated for the focal plane does not apply outside of the focal plane in the presence of objective misalignment. It is worth noting that a reduction in the size of the apparent lateral spacing results from an increase in the physical field of view. Therefore, for the case when the image of the scan mirror is formed after the physical aperture of the objective, the field of view will increase with depth in the specimen (Figure 6, right). The apparent spacing between fabrication points will then appear to decrease with increasing depth.

**Figure 7:**
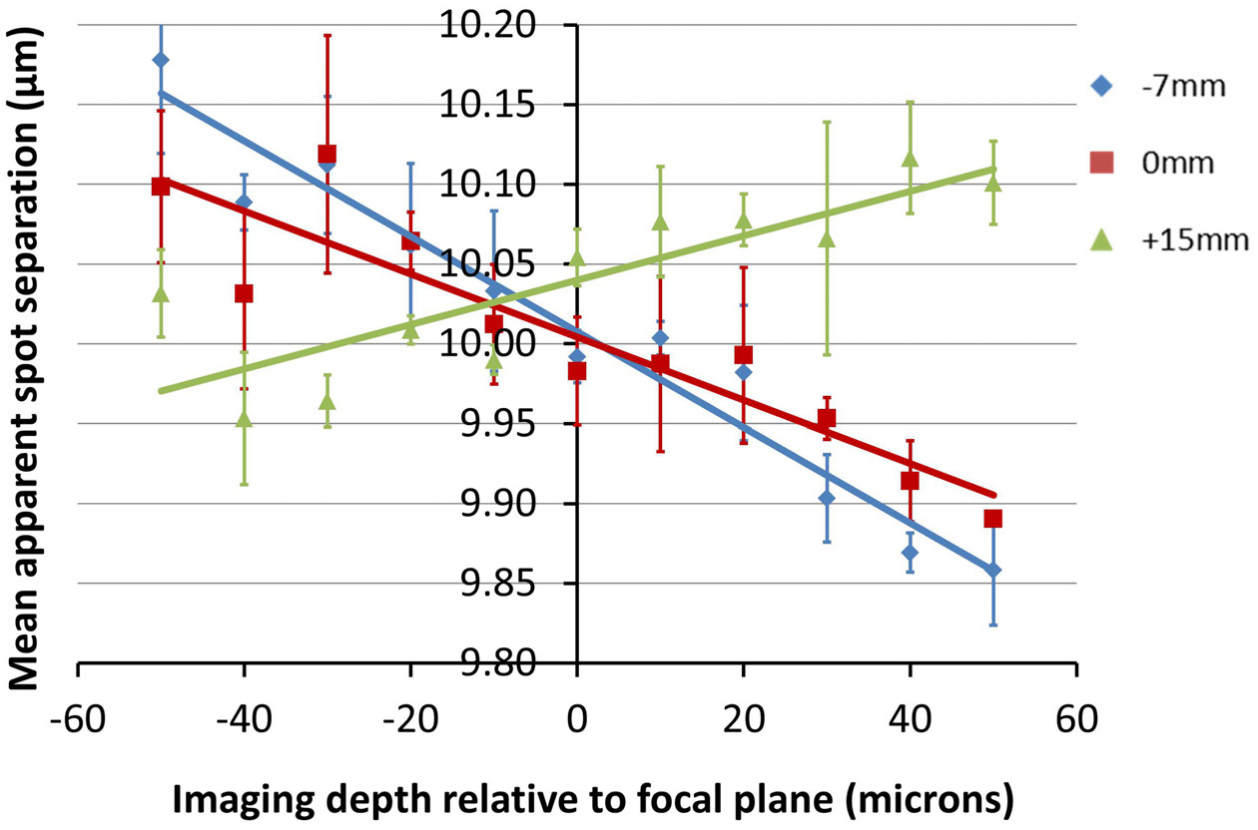
**Plots showing the change in apparent fabrication point separation as a function of depth for the objective locations (*Z*_0_-values) indicated**. The average lateral feature separation was calculated five times for each image, with the average and standard deviation value shown for each point. Positive values of depth indicate a displacement toward the objective.

The data shown in Figure 7 is in keeping with the predictions of the ZEMAX simulation. Here we can see that for all three positions of the objective lens, the measured feature separation is most accurate at the focal plane, with errors of 0.5% or less. Apparent changes in magnification of 3% over the depth range can be seen for *Z*_0_ = −7 mm. This is equivalent to a displacement of 13 μm at the edge of a 450 μm field of view. Error bars were calculated by processing each image five times and determining the standard deviation of the mean separation value.

**Figure 8:**
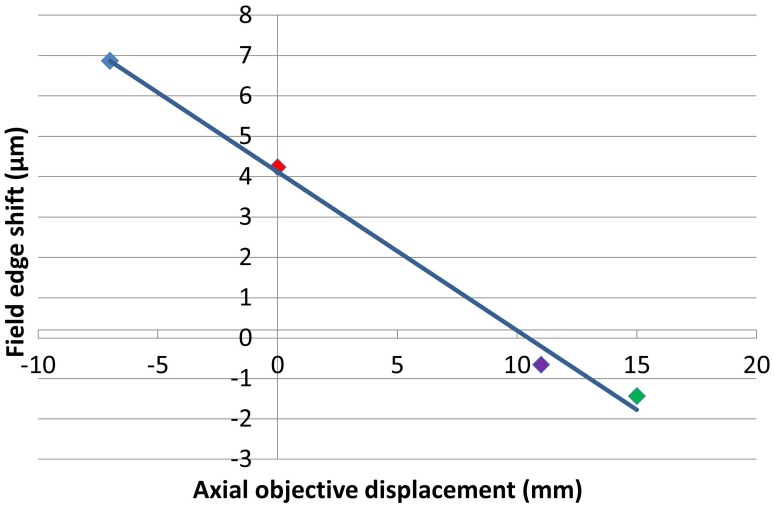
**Plot illustrating the apparent change in position of a static point located at the edge of a 450 μm field of view between the top and bottom of a 100 μm stack for each of the objective positions shown**. The colors of the points correspond to *Z*_0_-values of −7 mm (blue), 0 mm (red), 11 mm (purple), and 15 mm (green).

The exact location of the ideal objective position can be determined by first calculating the magnification gradient for each lens position and then interpolating between these positions to find a point of zero gradient (Figure 8). This gives a new best estimate for the axial location at *Z*_0_ = 11 mm. This is calculated to the nearest millimeter owing to the limited precision with which the microscope stage can be adjusted to meet the new lens position. An image stack of the calibration specimen was taken at *Z*_0_ = 11 mm and the magnification gradients were calculated (Figure 8 and Table 1). This demonstrates that by taking images of the calibration specimen at just three positions it is possible to identify uniquely the optimal axial alignment of the objective, and reduce the field edge shift from over 7 μm at to less than 1 μm.

**Table 1:** **The apparent magnification at imaging depths of +50 μm and −50 μm relative to the focal plane for each of the objective lens positions**.

**Objective position (*Z*_0_) (mm)**	**Magnification at *Z* = +50 μm**	**Magnification at *Z* = −50 μm**	**Magnification gradient (m^−1^)**	**Field edge shift (μm)**
−7	1.0186	0.9866	320	7.11
0	1.0116	0.9907	208	5.06
+11	0.9984	1.0023	−39	−0.75
+15	0.9978	1.0046	−69	−1.19

*The magnification gradient (magnification change per meter) for each lens position and the field edge shift is also shown. The measured values for the interpolated lens position (Z_0_ = 11 mm) is also shown for comparison*.

### Application to cardiac imaging data

In the previous section we showed how it is possible to identify an optimal objective location for the acquisition of distortion-free images with a remote focussing microscope. In many cases in microscopy it is not always possible to fix the objective location. For example, when imaging live cardiac tissue (see Section Cardiac Imaging), the limited range of the stage height adjustment made it necessary to bring the objective to the lowest extent of the focussing range (*Z*_0_ = −7 mm) in order to bring the tip into direct contact with the tissue surface. Whilst this necessarily introduces distortions, if the distortions are known then they can be compensated for in post-processing.

Figure 8 shows the relationship between the degree of distortion and objective location for the specific 0.8 NA water-immersion objective used to image the relatively uniform plastic calibration specimen. As described above, imaging in the lower refractive index heterogeneous cardiac tissue data was achieved with a 1.15 NA water-immersion objective. Given the difference in specimen composition, together with the capacity for stronger lenses to introduce greater degrees of distortion, there is no reason to expect the magnification gradients shown in Figure 7 to apply to the cardiac images and another means of obtaining the magnification gradient is required.

**Figure 9:**
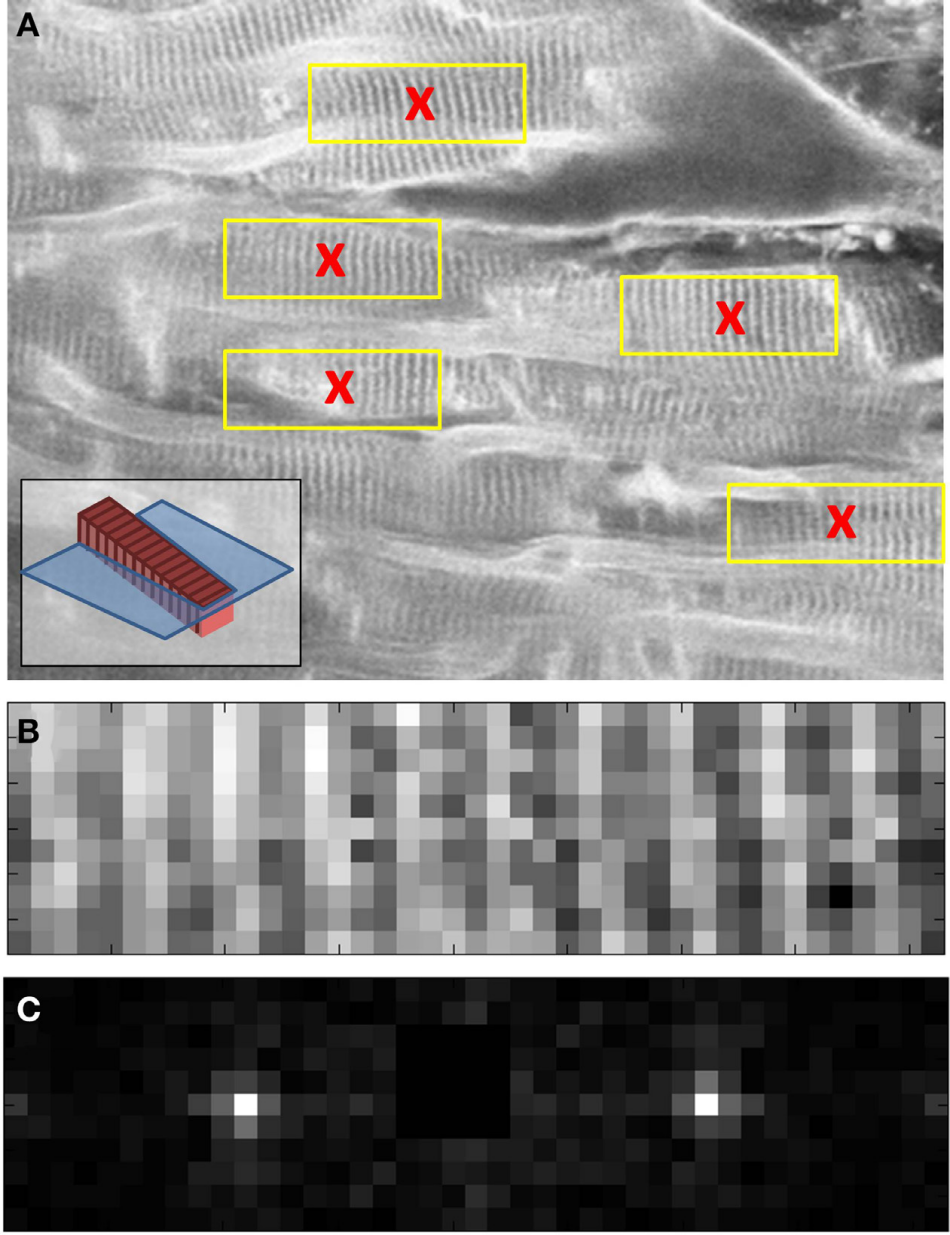
**Images taken from three different stages of raw data processing to determine the average SL of cells at a given depth of living cardiac tissue**. A two-photon XY image (close up shown in **A**) is used to identify several cardiomyocytes cells with clearly identifiable sarcomeres (red crosses in **A**). To aid interpretation of the images, the inset in **(A)** shows a model cell (red) with sarcomeres (dark red) inclined with respect to the focal plane (blue). 40 × 10 pixel sub-images (yellow boxes in **A** and close up in **B**) are taken from each of the identified cell candidates in the two-photon image. **(C)** A Fourier transform is used together with image metadata to identify the mean SL of each cell. The centroid of the Fourier space peak in **(C)** was calculated using a 5 × 5 pixel window centered on the peak. The centroid value was then used together with the real space image size (in μm) to determine the SL. See main text for full details.

In the absence of a suitably index-matched calibration specimen, a new approach to obtaining the magnification gradient associated with the objective location was required for the cardiac data. It was decided that the data itself could be used to provide a coarse estimate of the image distortion. The SL was used as a measure of a fixed separation. As the SL is expected to vary slightly with the orientation of the cell (see for example Botcherby et al., 2013) measurements were taken from at least three cells within the field of view at each depth and the average SL taken (Figure 9A).

**Figure 10:**
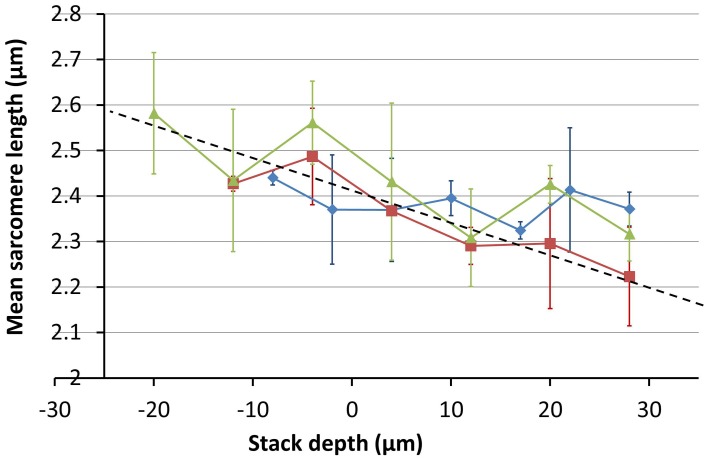
**Measurements of mean SL as a function of imaging depth**. Measurements were made of at least three cells at each depth. Images were taken at three different sites across two different hearts; one SD and one SHR (red = SHR, green and blue = SD). The trend line shows a steep change in image magnification with imaging depth.

To determine the SL measurements for the cells within the field of view, a semi-automated algorithm was implemented in MATLAB (The Mathworks Inc.). First, the user selected cells of interest within the field of view of each image in the stack (Figure 9A). A 10 × 40 pixel window, centered on each selected point, was used to create a sub-image of the cell (Figure 9B). The Fourier transform of this sub-image was then taken (Figure 9C). The centroid of the Fourier space peak (Figure 9C) was calculated using a 5 × 5 pixel window centered on the peak. The centroid value was then used together with the cell sub-image size (in μm) to determine the SL. The average and standard deviation of these SL values were then used to plot the points shown in Figure 10.

The variation in the mean SL with depth was obtained from image stacks taken from three different sites across two different hearts; one SD and one SHR. The results shown in Figure 10 indicate that the change in magnification is around 20% between the top and bottom of the 80 μm image stack, much greater than the 3% obtained for the calibration specimen at the same objective position. The absolute values of the SL are larger than the range of 1.9 μm to 2.1 μm reported in the literature for intact heart tissue (Bub et al., 2010). This is largely due to the enlargement of the Langendorff-perfused heart once removed from the chest cavity (edema) and the longer time to first image acquisition (owing to the physical separation of the tissue preparation and imaging labs).

**Figure 11:**
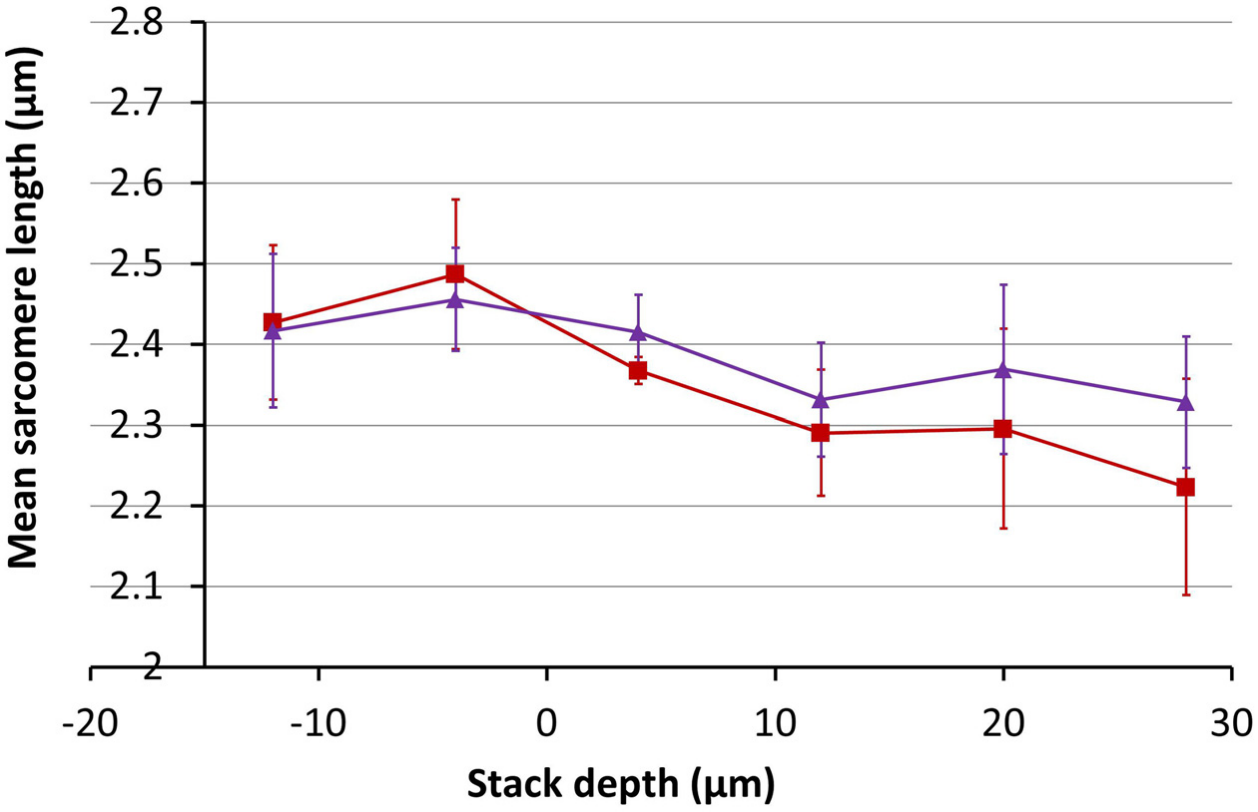
**Measurements of the mean SL as a function of depth in SD rodent hearts before (red) and after (purple) correction for a 20% change in magnification with depth**.

**Figure 12:**
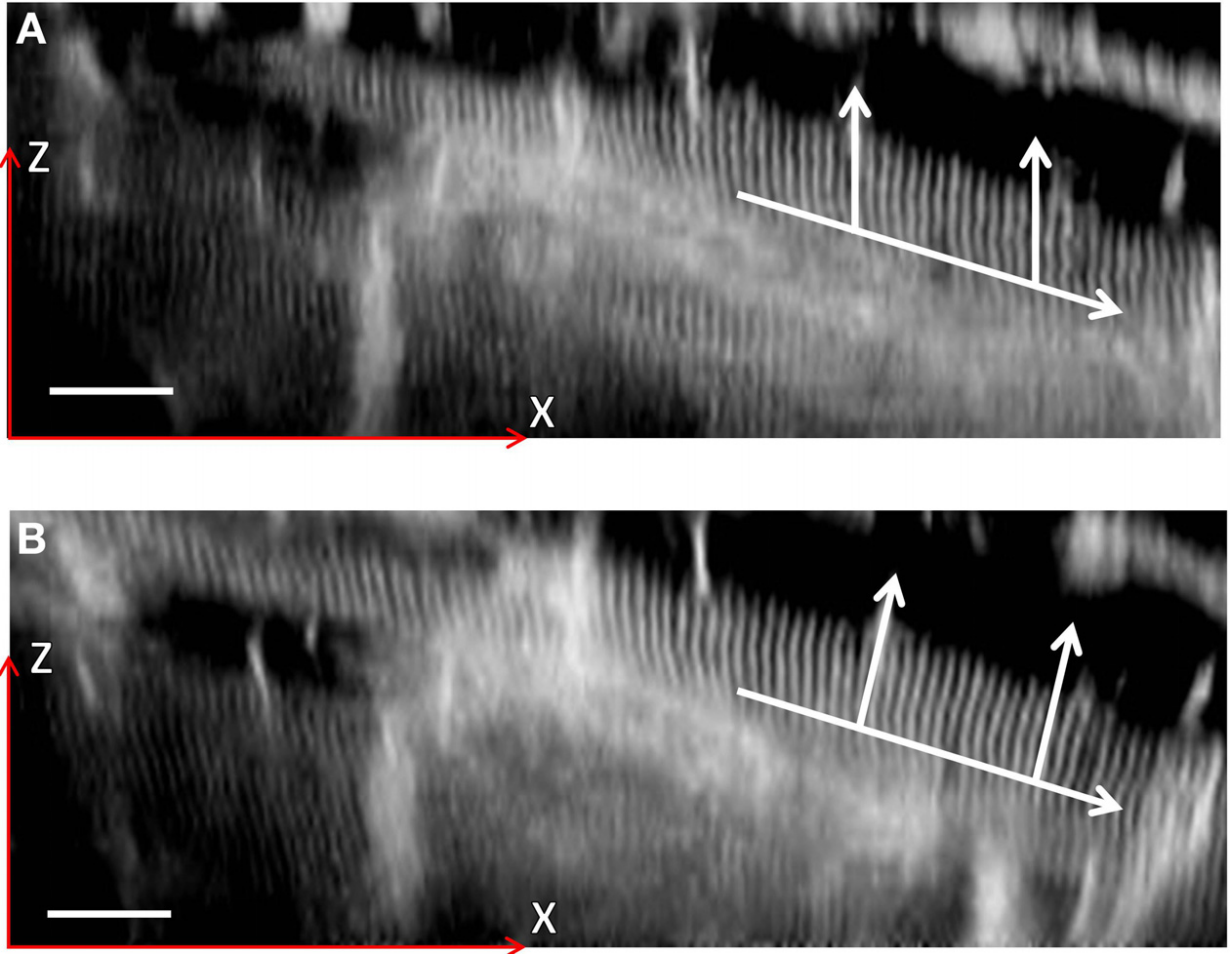
**XZ sections of a two-photon 3D image stack**. A single cardiomyocyte is shown highlighted both before **(A)** and after **(B)** a coarse distortion correction. The lower arrow indicates the direction of the cell axis, with the vertical arrows indicating the direction of the sarcomeres. The image contrast has been enhanced to highlight the sarcomere structure. Scale bar: 20 μm in the focal plane (located at half the height of the image).

Using the magnification change of 20% obtained from Figure 10, the inverse correction was applied to the 3D data set obtained from the SD heart tissue. Measurements of the average SL before and after correction as a function of depth are shown in Figure 11. After application of the data correction, the variance of the mean SL measurement across the depth range shown fell by nearly 50%, from 0.098 to 0.051 μm. Visualizing the imaging volume (Volume Viewer plugin for ImageJ) before and after correction revealed some additional features of the correction. Figure 12 shows an XZ image of a cardiomyocyte cell oriented on an incline. Before correction the sarcomeres do not lie orthogonal to the cell axis but are closer to vertical. After correction, the sarcomeres appear oriented closer to their natural direction, orthogonal to the cell axis. The before and after images are not quite identical as magnification changes along the Y-axis will significantly change the appearance of two otherwise equivalent planes in the raw and corrected data sets. In this example, the XZ cross-section is taken close to the edge of the field of view, where the Y-axis scaling will be most noticeable.

Whilst providing a useful illustration, the images in Figure 12 do not by themselves provide proof of perfect distortion compensation and more data is required to accurately constrain the true magnification range seen in the cardiac tissue images. At the same time, the 20% magnification change indicated in Figure 10 highlights the importance of accurate objective alignment before obtaining physiologically relevant data from remote focussing images.

## Discussion

Remote focussing microscopy is increasingly being adopted as a valuable biomedical research tool. To date, little work has been done to determine the alignment tolerance of the reference and imaging objectives, and the impact this has on the imaging volume. The work reported here offers an empirical approach to identifying an optimal alignment that avoids the need to track the precise location of conjugate image planes throughout a complex optical system. This approach takes advantage of developments in laser writing to develop a unique volumetric calibration tool. The calibration tool offers the necessary levels of robustness and uniformity to allow subtle changes in magnification across a large (450 μm × 450 μm × 100 μm) image volume to be detected.

The sensitivity of the distortion measurement to the axial objective alignment is limited by a number of factors. A major practical limitation is in the positioning of the microscope stage. At present, changes in the objective position require a coarse adjustment of the stage height followed by a fine adjustment of the objective to bring the specimen into the focal plane. This limits the accuracy of the true objective displacement to the nearest millimeter. Further improvement in this accuracy may well be achieved with a motorized stage that will allow the specimen to be moved to within the focal plane of the objective without any fine adjustment of the objective position.

The signal to noise ratio of the calibration specimen images could be improved by adopting dyes which are better matched to the PMT sensitivity window and substrates with greater purity. This, together with more sophisticated image processing algorithms, would allow the fabrication features within the calibration specimen to be located with greater precision, reducing the variability of the feature separation distance. In the short-term this could be improved by imaging a smaller field of view to increase the number of sampling points across each micrometer-sized feature.

Comparing Figure 7 with Figure 10 demonstrates how the degree of distortion produced by an axial objective displacement is a strong function of the imaging parameters, particularly the numerical aperture of the objective, the immersion medium and the refractive index of the specimen. For most experiments, it is anticipated that the calibration specimen would be used to determine an ideal alignment, which would then be used to acquire distortion-free biomedical imaging data. However, if the imaging objective could not be aligned ideally, then the degree of imaging volume distortion specific to the biological tissue would be required. It is conceivable that this might be achieved with a calibration specimen having a refractive index closer to water. Whilst this might be possible with certain types of aerogels, any deformable substrate would need to be tested for stability and repeatability.

At present, the estimate of the distortion within the whole heart data is limited by the number of data points. Confidence in the estimated magnification range of 20% over 80 μm of depth would improve with the number of data sets analyzed. However, even if the true magnification range is only half this figure, there would still be a strong case for employing measures to ensure the fidelity of the imaging data acquired with remote focussing microscopes. Given the size of the distortion in biological tissue and the difficulties in achieving an accurate correction post-acquisition, the best advice would appear to be to fix the objective in the ideal imaging location first and then image. The calibration specimen presented in this paper allows this ideal objective position to be uniquely determined, without requiring complicated alignment procedures. Whilst calibration protocols are not new to microscopy, they are typically restricted to two-dimensions. Here we present a potential solution to the problem of three-dimensional calibration for the next generation of optical microscopes.

## Conclusion

We have demonstrated the use of a volumetric calibration specimen to determine the optimal axial alignment of an objective lens in a remote focussing microscope. The calibration specimen allowed the axial change in magnification predicted in simulation to be mapped out for three different positions of the objective lens. Interpolation between these three positions allowed the optimal (telecentric) position of the objective to be uniquely identified. The accuracy of the technique was validated by demonstrating the reduction of image distortion from over 7 μm of displacement error at the edge of the field of view to less than 1 μm.

The distortion of the image volume was first estimated and then compensated in a 3D stack of two-photon images of living cardiac tissue. By assuming the average SL at a given depth to have a fixed value, the change in magnification was measured to be as much as 20% over the 80 μm depth range of the 3D stack. Compensating for this magnification change in post-processing was seen to nearly halve the variance of the measured SL value.

### Conflict of interest statement

The authors declare that the research was conducted in the absence of any commercial or financial relationships that could be construed as a potential conflict of interest.

## Abbreviations

SL: Sarcomere length
SD: Sprague-Dawley rats
WKY: Wistar-Kyoto rats
SHR: Spontaneously hypertensive rats.
